# Expression of Piezo1 in the Trigeminal Neurons and in the Axons That Innervate the Dental Pulp

**DOI:** 10.3389/fncel.2022.945948

**Published:** 2022-06-29

**Authors:** Yi Sul Cho, Hye Min Han, Soon Youn Jeong, Tae Heon Kim, So Young Choi, Yun Sook Kim, Yong Chul Bae

**Affiliations:** ^1^Department of Anatomy and Neurobiology, School of Dentistry, Kyungpook National University, Daegu, South Korea; ^2^Department of Oral & Maxillofacial Surgery, School of Dentistry, Kyungpook National University, Daegu, South Korea

**Keywords:** Piezo1, mechanosensitive channel, dentin sensitivity, ultrastructure, dental pulp

## Abstract

Information on the neurons and axons that express the mechanosensitive channel Piezo1 and its expression in axons innervating the dental pulp may help understand the nature of the Piezo1-mediated mechanosensation and the underlying mechanism of dentin sensitivity elicited by mechanical stimuli. For this, we here investigated the neurochemical properties of the neurons in the rat trigeminal ganglion (TG) and their axons in its sensory root that express Piezo1 and the expression of Piezo1 in the rat and human dental pulp by light and electron microscopic immunohistochemistry and quantitative analysis. Piezo1 was expressed mainly in medium-sized and large TG neurons. Piezo1-immunopositive (+) neurons frequently coexpressed the marker for neurons with myelinated axons, NF200, but rarely the markers for neurons with unmyelinated axons, CGRP or IB4. In the sensory root of TG, Piezo1 was expressed primarily in small myelinated axons (Aδ, 60.2%) but also in large myelinated (Aβ, 24.3%) and unmyelinated (C, 15.5%) axons. In the human dental pulp, Piezo1 was expressed in numerous NF200+ axons, which formed a network in the peripheral pulp and often “ascended” toward the dentin. Most Piezo1+ myelinated axons in the radicular pulp became unmyelinated in the peripheral pulp, where Piezo1 immunoreaction product was associated with the axonal plasma membrane, suggesting a functional role of Piezo1 in the peripheral pulp. These findings suggest that Piezo1 is involved primarily in mediating the acute pain elicited by high-threshold mechanical stimuli, and that the Piezo1-mediated dental mechanotransduction occurs primarily in the axons in the peripheral pulp.

## Introduction

The Piezo channel family members, Piezo1 and Piezo2, are nonselective cation channels that are activated by mechanical stimuli (Wu et al., 2017a,b). Piezo2 is expressed primarily in nervous system tissues, such as neurons in the trigeminal ganglion (TG; Won et al., 2017), dorsal root ganglia (DRG), and Merkel cells (Woo et al., 2014; Shin et al., 2021), whereas Piezo1 is expressed primarily in nonneuronal tissues, such as blood vessels and cells in the urinary tract that are exposed to fluid flow (Wu et al., 2017b; Douguet et al., 2019; Dalghi et al., 2021). Piezo2 is activated primarily by low-threshold mechanical stimuli like touch, and it is expressed predominantly in peripheral Aβ fibers (Ranade et al., 2014; Woo et al., 2014). It is also implicated in the mechanisms of acute and pathologic mechanonociception associated with Aδ and C fibers (Nencini and Ivanusic, 2017; Murthy et al., 2018). Recently, Piezo1 was also found in neurons in the DRG and TG, and experiments with administration of the specific Piezo1 agonist Yoda1 into peripheral tissues suggested involvement of Piezo1 in the pain elicited by mechanical stimuli (Mikhailov et al., 2019; Wang et al., 2019; Della Pietra et al., 2020; Roh et al., 2020). However, the type of peripheral axon that expresses Piezo1 remains unknown; identifying it may help elucidate the nature of the Piezo1-mediated mechanosensation, including its potential involvement in low-threshold mechanoreception, acute nociceptive, and pathological pain.

Dentin sensitivity is perhaps the most frequent form of dental pain. The widely accepted hydrodynamic theory of dentin sensitivity states that various stimuli, such as air blasts or probing applied to the dentin surface, cause movement of fluid in the dentinal tubules and the peripheral pulp, which activates nearby axons and elicits pain (Brännström and Aström, 1964; Charoenlarp et al., 2007; Vongsavan and Matthews, 2007). This theory assumes the presence of a mechanoreceptor in the axons in the dentinal tubules and the peripheral pulp. However, so far, there have been no studies reporting the expression of mechanoreceptors in the axons of this part of the pulp.

To address this, we investigated the neurons in the TG and axons in its sensory root and the axons within the dental pulp that express Piezo1 by light and electron microscopic immunohistochemistry and quantitative analysis.

## Materials and Methods

All experimental procedures involving animals were approved by the Kyungpook National University Intramural Animal Care and Use Committee and were performed according to the guidelines of the National Institute of Health. All human material was collected only after the nature of the experiments was explained to the patients, and they signed the appropriate informed consent forms that have been approved by the Research and Ethics Committee of the Kyungpook National University Dental Hospital.

Material from seven 9-week-old male Sprague-Dawley rats weighing 300–320 g and six healthy maxillary premolar teeth, extracted during orthodontic treatment from five 16–28-year-old male patients, was used for this study. Four rats were used for light microscopic (LM) immunohistochemistry, and three rats were used for electron microscopic (EM) immunohistochemistry; three human teeth were used for LM immunohistochemistry, and three teeth were used for EM immunohistochemistry.

### Tissue Preparation

The rats were deeply anesthetized using sodium pentobarbital (80 mg/kg, i.p.) and perfused through the heart with a freshly prepared fixative containing 4% paraformaldehyde (PFA) in 0.1 M phosphate buffer (PB, pH 7.4) for LM immunohistochemistry and a mixture of 4% paraformaldehyde and 0.01% glutaraldehyde in 0.1 M PB for EM immunohistochemistry. The right trigeminal ganglion and its proximal sensory root, and the dental pulp of the right maxillary 1st and 2nd molars were carefully removed and preserved for LM (TG, dental pulp) and EM (proximal sensory root) immunohistochemistry. The human teeth were sectioned longitudinally with a water-cooled high-speed diamond bur, and the pulps were carefully removed and preserved for LM and EM immunohistochemistry. Then, the specimens were cryoprotected in 30% sucrose in PB at 4°C overnight. The next day, the rat TGs and dental pulps and human dental pulps were cut on a freezing microtome at 40 μm for LM, and the sensory roots of the rat TG and the human dental pulps were cut transversely on a vibratome for EM and stored in PB at 4°C.

### Light Microscopic Immunohistochemistry

For immunofluorescence, sections of rat TGs and dental pulps and human dental pulps were treated with 50% ethanol in 10% normal donkey serum (NDS, Jackson ImmunoResearch, West Groove, PA) for 30 min and incubated overnight in a rabbit anti-Piezo1 antiserum (1:300; APC087, Alomone Labs, Jerusalem) alone or in combination with mouse anti-calcitonin gene-related peptide (CGRP, 1:1,000; ab81887, Abcam, Cambridge, MA), fluorescein isothiocyanate-conjugated isolectin B4 (FITC-conjugated IB4; 1:1,000; L2895, Sigma-Aldrich, St. Louis, MO), or mouse anti-NF200 (1:20,000; N0142, Sigma-Aldrich) antisera. On the next day, the sections were incubated with a Cy3-conjugated donkey anti-rabbit antibody alone or in combination with the FITC-conjugated donkey anti-mouse antibody (1:200, in PB, Jackson ImmunoResearch) for 3 h. Sections were mounted on slides, coverslipped with Vectashield (Vector Laboratories, Burlingame, CA), and examined on a Zeiss Axioplan 2 fluorescence microscope or LSM 510 Meta confocal microscope (Carl Zeiss, Gottingen, Germany).

### Electron Microscopic Immunohistochemistry

Electron microscopic immunohistochemistry was performed according to the method previously published from our laboratory (Bae et al., 2015, 2018). Briefly, sections of the sensory root of the rat TG and human dental pulp were incubated with 3% H_2_O_2_ and blocked with 10% NDS. Then, sections were incubated with the rabbit anti-Piezo1 antibody at 1:300 in PBS overnight. On the next day, sections were incubated with 2% NDS for 10 min and then with a donkey anti-rabbit antibody at 1:200 for 2 h. Avidin-biotin-peroxidase binding was with ExtrAvidin peroxidase (1:5,000; Sigma-Aldrich) for 1 h; immunoperoxidase was visualized with DAB. After treating with 1% osmium tetroxide in PB for 1 h, the sections were dehydrated in a series of ethanol dilutions and embedded in Durcupan ACM (Fluka, Buchs, Switzerland) and then cured at 60°C for 48 h. Small chips of the embedded tissue were cut out and glued onto blank resin blocks. Thin sections were cut and stained with uranyl acetate and lead citrate according to standard protocols. The grids were examined with a Hitachi H 7500 electron microscope (Hitachi, Tokyo, Japan) at 80 kV, and images were captured at 8,000 × , 10,000 × , or 25,000 × original magnification using an SC1000 CCD camera (Gatan, Pleasanton, CA) and saved as TIFF files.

### Quantitative Analysis

Light micrograph images at 200 × original magnification (857 × 652 μm^2^) from the opthalmomaxillary area of the TG in 2–3 sections in each of four TGs (a total of 8–12 sections) were obtained with an Exi camera (Q-Imaging Inc., Surrey, CA) attached to a Zeiss Axioplan 2 microscope and saved as TIFF files. The cross-sectional area of the Piezo1+ neuronal cell bodies with clearly visible nucleoli and the fraction of the Piezo1+ neurons that were also co-stained for CGRP, IB4, or NF200 were analyzed using the ImageJ software (NIH, Bethesda, MD). The Piezo1+ somata were divided into three size categories, namely, small (<500 μm^2^ in cross-sectional area), medium (500–1,000 μm^2^), and large (>1,000 μm^2^). Inter-animal variability in the cross-sectional area of neuronal cell bodies from different ganglia was not significant (one-way ANOVA), so the data could be pooled for analysis.

The cross-sectional area of all Piezo1+ axons within a field of 10,000 μm^2^ in each of two sections of the proximal (peripheral) sensory root of the TG from each of 3 rats was analyzed. The Piezo1+ axons were divided into three types, namely, unmyelinated, small myelinated (<20 μm^2^ in cross-sectional area, corresponding to <5 μm in diameter), and large myelinated (>20 μm^2^ in cross-sectional area, corresponding to >5 μm in diameter), corresponding to C, Aδ, and Aβ fibers, respectively (Debanne et al., 2011; Boron and Boulpaep, 2012).

The fractions of the Piezo1+ myelinated and unmyelinated axons of all Piezo1+ axons were analyzed on electron micrographs taken from the radicular pulp, the core of the coronal pulp, and the peripheral pulp within a field of 10,000 μm^2^ in each of two sections from each pulp region of three human teeth.

The fractions of Piezo1+ axons in the human dental pulp that coexpress CGRP or NF200 were analyzed in 9–12 confocal images from 3 to 4 sections of each of the coronal and the peripheral regions of three human dental pulps. Images were captured with an LSM 510 Meta confocal microscope (Carl Zeiss) at a magnification of 40 × (225 × 225 μm) at the same optical slice thickness for all channels and saved as TIFF files. All axons longer than 1 cm in the images (corresponding to longer than 45 μm in tissue) were counted; multiple axonal segments in a linear arrangement that appeared to belong to the same axon were counted as a single axon.

The data were first assessed for a normal distribution using the Shapiro–Wilk test to determine the use of parametric or nonparametric statistical analysis. All variables were normally distributed (*p* > 0.05), and thus, parametric tests were used as follows. The differences in the fraction of each Piezo1+ axon type and in the proportion of Piezo1+ myelinated and unmyelinated axons among the three pulpal regions were examined by one-way analysis of variance (ANOVA) and Scheffe's F-test. The difference between the fractions of Piezo1+ axons that coexpress NF200 and CGRP was examined using the unpaired Student's *t*-test; significance was set at *p* < 0.05.

### Immunohistochemical Controls

**Figure 1:**
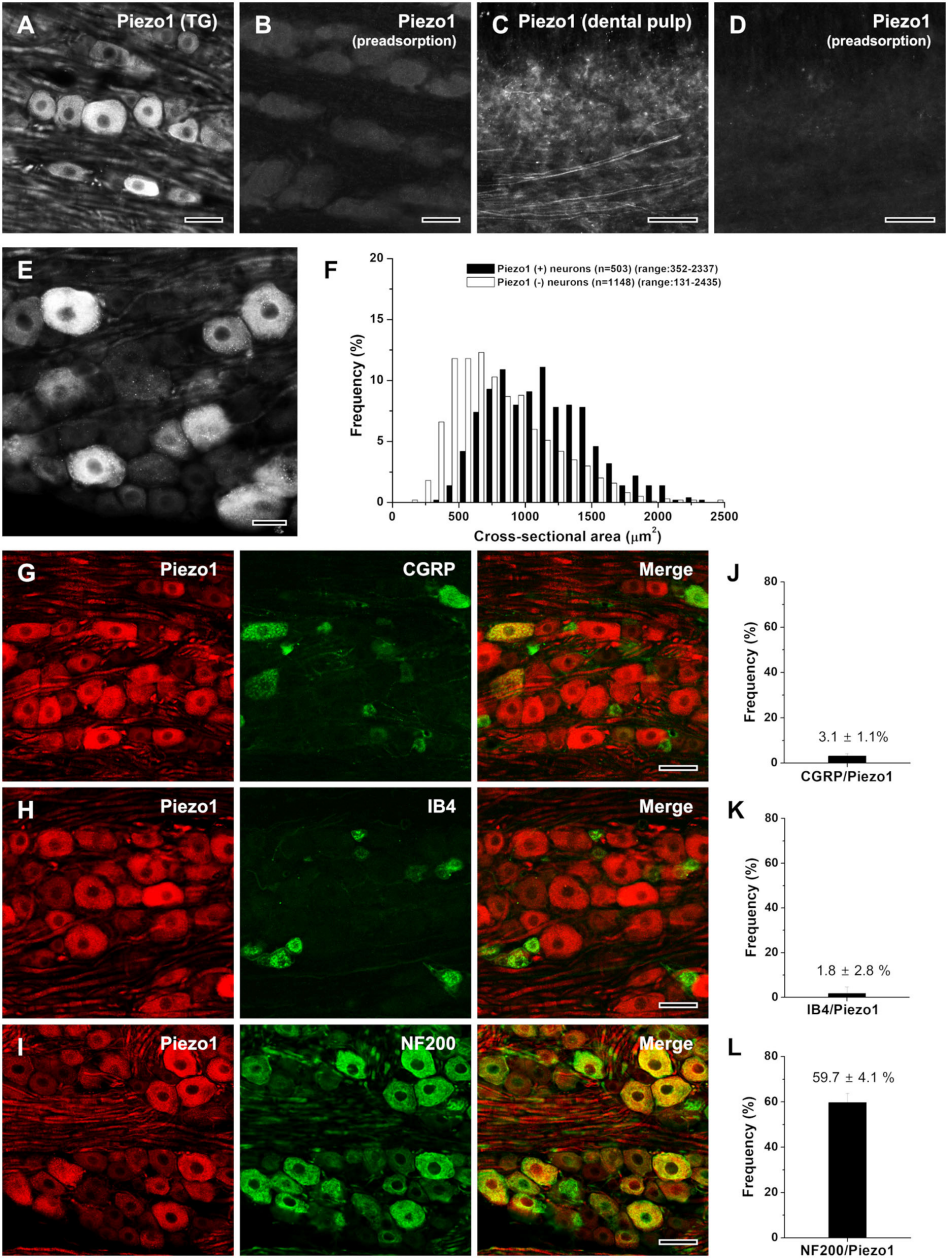
Light micrographs showing the specificity of Piezo1 immunostaining in the rat trigeminal ganglion [TG; **(A,B)**] and the human dental pulp **(C,D)**, the size distribution **(E,F)**, and the neurochemical characterization **(G–L)** of Piezo1+ neurons in the rat TG. **(A–D)** Immunofluorescent staining for Piezo1 in the rat TG **(A, B)** and the human dental pulp **(C,D)**. The Piezo1 immunostaining in the TG neurons and pulpal axons is completely abolished by preadsorption with the corresponding blocking peptide, confirming the specificity of the Piezo1 antibody. **(E,F)** Light micrograph showing Piezo1+ neurons in the TG **(E)** and their size distribution **(F)**. Piezo1 is expressed mostly in medium-sized and large neurons. **(G-L)** Double immunofluorescent staining for Piezo1 and CGRP **(G)**, IB4 **(H)**, or NF200 **(I)**, and the quantitative analysis of colocalization of Piezo1 and CGRP **(J)**, IB4 **(K)**, or NF200 **(L)**. Piezo1+ neurons frequently co-stain NF200 but seldomly for CGRP or IB4. Scale bars = 50 μm in **(A–E, G–I)**.

To control for the specificity of the Piezo1 antibody, we processed tissues according to the above protocols, except that either the anti-Piezo1 antibody was omitted or a Piezo1 blocking peptide (Piezo1: BLP-PC087, Alomone Labs) was added following the recommendation of the manufacturer. Specific immunostaining was completely abolished by omission of the antibody or preadsorption with the Piezo1 blocking peptide at a final concentration of 8 μg/ml (Figures 1A–D).

## Results

### Trigeminal Neurons That Express Piezo1

About 30% (503/1,651) of the neurons in the opthalmomaxillary area of the TG were Piezo1-immunopositive (+), similar to the fraction of DRG neurons that respond to the Piezo1 agonist Yoda1 (Mikhailov et al., 2019; Roh et al., 2020). Piezo1 was expressed mostly in large- and medium-sized neurons (>500 μm^2^ in cross-sectional area, 91.9%) and rarely in small neurons (<500 μm^2^, 8.1%; Figures 1E,F). Double immunofluorescent staining revealed that Piezo1+ neurons frequently coexpressed the marker for neurons with myelinated fibers, NF200 (59.7 ± 4.1%), but only occasionally coexpressed the marker for peptidergic neurons with unmyelinated fibers, CGRP (3.1 ± 1.1%) or the marker for non-peptidergic neurons with unmyelinated fibers, IB4 (1.8 ± 2.8%; Figures 1G–L).

### Axons in the Sensory Root of the TG That Express Piezo1

**Figure 2:**
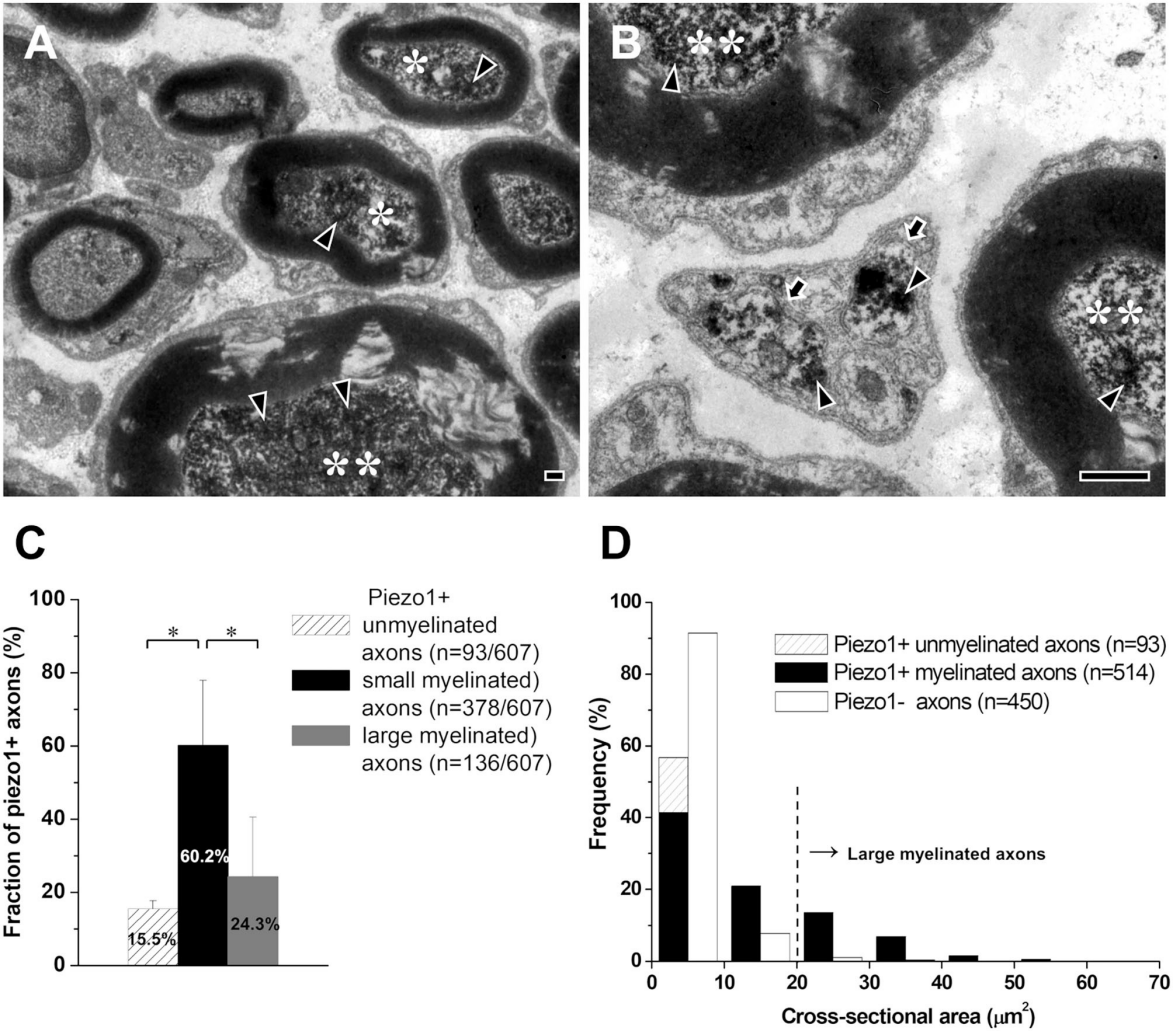
Axons that express Piezo1 in the sensory root of the rat trigeminal ganglion. **(A,B)** Electron micrographs showing immunoreaction products for Piezo1 (arrowheads) in small myelinated axons (asterisks), large myelinated axons (double asterisks), and unmyelinated axons (arrows). **(C,D)** Histograms showing the fraction **(C)** and the size distribution **(D)** of the Piezo1+ small myelinated, large myelinated, and unmyelinated axons. Asterisks in **(C)** indicate significant differences (*P* < 0.05) between the fraction of small myelinated axons and that of large myelinated or unmyelinated axons. Scale bars = 500 nm.

We studied the type (unmyelinated, small myelinated, and large myelinated) of axons that express Piezo1 in the proximal (peripheral) sensory root of the TG that contains only axons, close to their origin, because myelinated axons usually lose their myelin sheath during their peripheral course to the target tissue (Peng et al., 1999; Paik et al., 2010). The majority (60.2% ± 17.7%) of the Piezo1+ axons were small myelinated (<20 μm^2^ in cross-sectional area, corresponding to Aδ fibers), 24.3 ± 16.3% were large myelinated (>20 μm^2^, corresponding to Aβ fibers), and 15.5 ± 2.2% were unmyelinated (corresponding to C fibers, Figures 2A–D).

### Axons in the Dental Pulp That Express Piezo1

**Figure 3:**
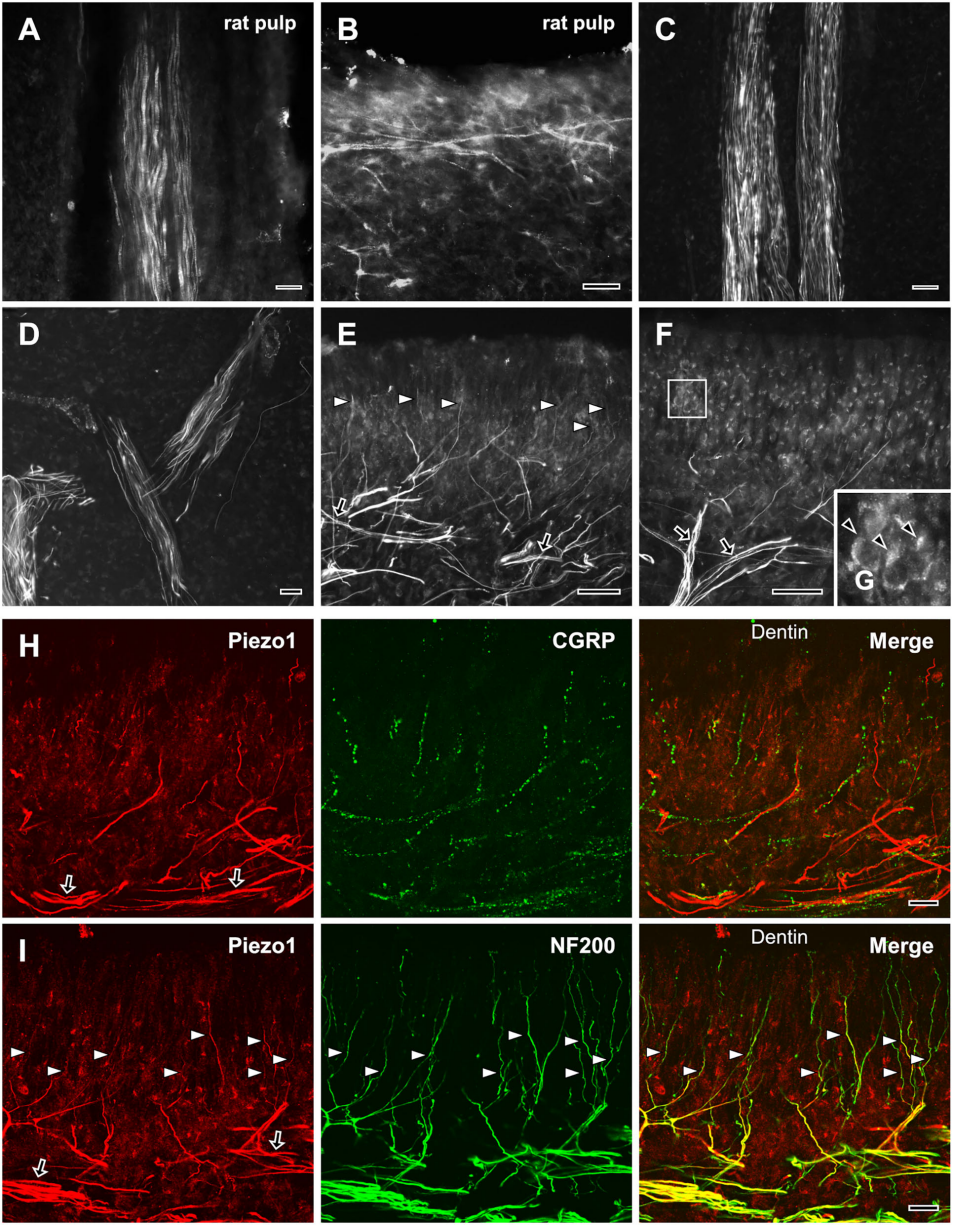
Light micrographs of immunofluorescent staining for Piezo1 in axons in the rat **(A,B)** and the human dental pulp **(C–G)**, and double immunofluorescent staining for Piezo1 and CGRP **(H)** or NF200 **(I)** in the human dental pulp. **(A,B)** Piezo1 is expressed in a few axons in the rat radicular **(A)** and coronal **(B)** pulp. **(C–G)** Piezo1 is expressed in a few axons in the human radicular pulp **(C)** and the core of the coronal pulp **(D)**, but in numerous axons (arrows) that branch extensively in the peripheral pulp **(E)**, and in many axons “ascending” toward dentinal tubules (arrowheads in **E**). Piezo1 is also expressed in odontoblasts in the peripheral pulp [arrowheads in **(G)**, **(G)** is an enlargement of the boxed area in **(F)**]. **(H,I)** Double immunofluorescent staining for Piezo1 and CGRP **(H)** or NF200 **(I)** in the peripheral region of the human dental pulp; colocalization is represented in yellow. Piezo1 is expressed mostly in NF200+ axons that form a plexus in the peripheral pulp (arrows) and “ascend” toward the dentin (arrowheads). Scale bars = 20 μm.

Piezo1 was expressed in axons innervating the rat (Figures 3A,B) and the human dental pulps (Figures 3C–I). The regional differences and the details of the distribution of Piezo1+ axons were more easily appreciated in the human pulp than in the rat pulp perhaps because of its much larger size. In the human dental pulp, Piezo1 was expressed in a small number of axons within the radicular pulp and the core of the coronal pulp (Figures 3C,D), but in a large number of axons in the peripheral pulp, forming a network (Figures 3E–I). It was also expressed in many axons “ascending” toward the dentinal tubules (Figures 3E,H,I). Double immunofluorescent staining showed that most of the Piezo1+ pulpal axons (82.7 ± 1.6%) coexpressed NF200, and a few (2.9 ± 0.7%) coexpressed CGRP (Figures 3H,I). In addition, Piezo1 was expressed in odontoblasts (Figures 3F,G), consistent with recent electrophysiological and immunohistochemical findings of Piezo1 in rat odontoblasts and human odontoblast-like cells (Sato et al., 2018; Sun et al., 2022).

**Figure 4:**
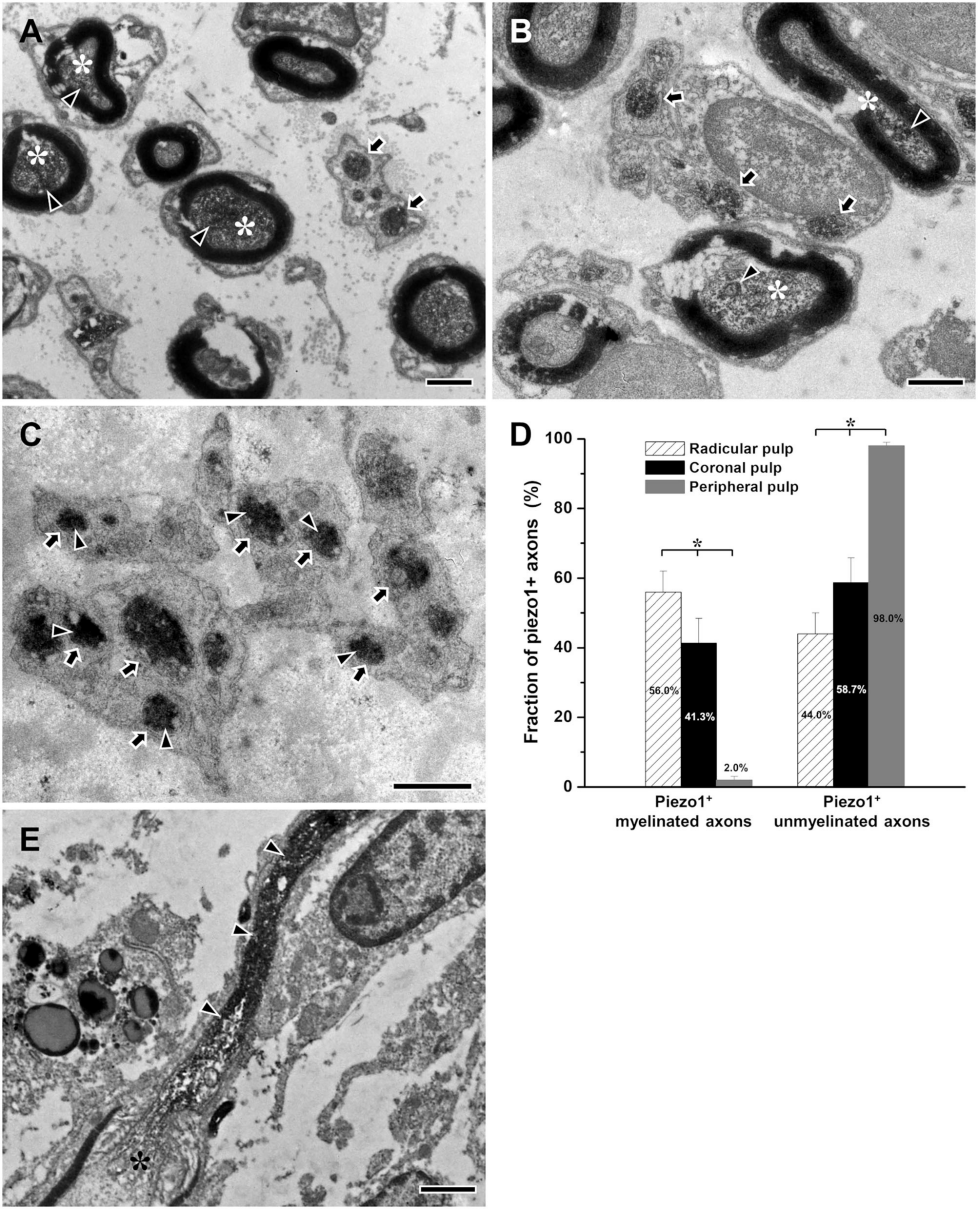
Electron micrographs showing immunostaining for Piezo1 (arrowheads) in myelinated (asterisks) and unmyelinated axons (arrows) in the radicular **(A)**, the core of the coronal **(B)**, and the peripheral **(C)** regions of the human dental pulp, and a histogram showing the fraction of Piezo1+ myelinated and unmyelinated axons of all Piezo1+ axons in each region of the human dental pulp **(D)**, and an electron micrograph of myelinated axon showing shedding of its myelin sheath during its course in the rat dental pulp [**(E)**, modified from Bae and Yoshida, 2020]. Piezo1+ myelinated axons are more frequent in the radicular pulp than in the core of the coronal pulp, and virtually all Piezo1+ axons are unmyelinated in the peripheral pulp, suggesting that most Piezo1+ myelinated axons in the radicular pulp lose their myelin and become unmyelinated in the peripheral pulp. This assumption can be supported by our previous study showing the shedding of myelin sheath from myelinated axon within the rat dental pulp **(E)**. Asterisks in **(D)** indicate significant differences (*p* < 0.05) among the fraction of Piezo1+ small myelinated or unmyelinated axons in each of the three regions of human dental pulp. Arrowheads in **(A–C)** indicate immunoreaction product of Piezo1. Arrowheads in **(E)** indicate unmyelinated portion of the myelinated axon (asterisk). Scale bar = 1 μm.

At the EM level, the electron-dense immunostaining for Piezo1 was observed in the axoplasm of pulpal axons; in the peripheral pulp, the immunoreaction product was near the axonal plasma membrane of unmyelinated axons, possibly visualizing a membrane-bound pool of the receptor (Figures 4A–C). The fraction of myelinated Piezo1+ axons of all Piezo1+ axons decreased significantly between the radicular pulp (55.9 ± 6.0%) and the peripheral pulp (2.0% ± 1.0%), whereas the fraction of unmyelinated Piezo1+ axons increased significantly from 44.1% ± 6.0 % in the radicular pulp to 98.0% ± 1.0% in the peripheral pulp (Figure 4D), suggesting that virtually all Piezo1+ myelinated axons in the radicular pulp lose their myelin and become unmyelinated at the peripheral pulp. This assumption can also be supported by a previous study, showing shedding of myelin sheath in myelinated axons in the rat dental pulp (Figure 4E, modified from Bae and Yoshida, 2020).

## Discussion

The main findings of this study are that Piezo1 is expressed primarily in small myelinated (Aδ) axons, suggesting its predominant involvement in mediating acute mechanical pain and that it is expressed in numerous unmyelinated axons in the peripheral pulp and axons “ascending” toward the dentinal tubules, suggesting that the axons in the peripheral pulp and the dentinal tubules are the sites of initiation of the Piezo1-mediated mechanotransduction.

### Expression of Piezo1 in the Trigeminal Ganglion and Its Sensory Root

Piezo1 was expressed in all types of axons, including large myelinated (Aβ), small myelinated (Aδ), and unmyelinated (C) axons. Considering that Aβ, Aδ, and C fibers that are activated by mechanical stimuli are primarily involved in low-threshold mechanoreception, acute mechanical pain, and mechanical hyperalgesia, respectively (Närhi et al., 1992; Ngassapa, 1996; Ahlgren et al., 1997; Fang et al., 2005), Piezo1 may be involved in all types of mechanosensation, including innocuous mechanosensation, acute nociceptive pain, and pathologic pain elicited by mechanical stimuli.

The fact that the majority of Piezo1+ axons are small myelinated (Aδ) suggests that Piezo1 in peripheral axons is involved primarily in the mechanism of acute nociceptive pain elicited by high-threshold mechanical stimuli. This is a role for neuronal Piezo1 that is very different from the one assumed heretofore, i.e., sensing shear stress by signaling low-threshold mechanical stimuli, such as fluid flow in the nonneuronal tissues (Douguet et al., 2019; Dalghi et al., 2021). It is also different from the predominant role of Piezo2 in sensing touch, and, consistently, predominant expression of Piezo2 in large myelinated (Aβ) fibers (Ranade et al., 2014; Woo et al., 2014). Indeed, in this study, about a quarter of the Piezo1+ axons were large myelinated, which are primarily low-threshold mechanoreceptors (Fang et al., 2005; Li et al., 2011; LTM), suggesting that in some peripheral axons, Piezo1 can be activated by LTM stimuli, like fluid flow or touch.

About 15% of Piezo1+ axons in the sensory root of the TG were unmyelinated. Since very few Piezo1+ neurons in the TG were immunopositive for CGRP (3.1%) or IB4 (1.8%), it is likely that the majority of the Piezo1+ afferent neurons that give rise to unmyelinated axons do not belong to the classical peptidergic and nonpeptidergic types of primary afferent neurons. If this is correct, it is also likely that the Piezo1-mediated mechanical hyperalgesia under pathologic conditions is transmitted *via* specific subsets of unmyelinated C axons (Wang et al., 2019). That only a small fraction of the Piezo1+ axons were unmyelinated is consistent with the observation that Piezo1 was expressed only occasionally in small neurons in the TG. However, it is at odds with the report that Piezo1 mRNA is expressed mostly in small DRG neurons (Wang et al., 2019); this discrepancy may be due to a difference or differences in the Piezo1 detection method (mRNA vs. protein), species (mice vs. rats), and tissues (DRG vs. TG).

This study did not perform functional study on the role of each Piezo1+ axon type. Morphological findings of Piezo1+ axons combined with functional data on the response of each Piezo1+ axon type to various mechanical stimuli in further studies can help understand the functional role of the Piezo1+ axons in the craniofacial area and in the dental pulp.

### Expression of Piezo1 in Axons Within the Dental Pulp

In the human dental pulp, Piezo1 was expressed in a few axons in the radicular pulp and the core of the coronal pulp, but in numerous unmyelinated axons in the peripheral pulp and axons “ascending” toward the dentinal tubules. Also, almost all the Piezo1+ myelinated axons in the radicular pulp lost their myelin sheath and became unmyelinated in the peripheral pulp, where Piezo1 immunoreaction product was associated with the axolemma of the unmyelinated fibers, suggesting that the receptor is membrane-bound and therefore functional in the peripheral pulp. These findings suggest that the axons in the peripheral pulp and the dentinal tubules are the principal sites of initiation of Piezo1-mediated mechanotransduction. They also provide morphological evidence supporting the hydrodynamic theory of dentin sensitivity, which is predicated on the presence of a mechanoreceptor in the axons in the peripheral pulp and the dentinal tubules, i.e., various stimuli applied to the dentin surface induce fluid flow in the peripheral pulp and in the dentinal tubules (Brännström and Aström, 1964; Matthews and Vongsavan, 1994; Charoenlarp et al., 2007), which activate mechanoreceptors on nearby axons to elicit pain. This is further consistent with (1) Piezo1 responds to shear stress by fluid flow (Wu et al., 2017a,b; Dalghi et al., 2021), (2) the peripheral pulp is innervated by a few large myelinated (Aβ) axons that are primarily low-threshold mechanoreceptors (Fang et al., 2005; Paik et al., 2009; Kim et al., 2017), and (3) the present data show that Piezo1 is expressed in many large myelinated (Aβ) axons. These findings also raise the notion that local application of a selective Piezo1 channel blocker to the peripheral pulp or the dentinal tubule can be effective for relieving dentin sensitivity elicited by dentinal fluid movement without the side effects of systemic application.

This study was performed on the trigeminal ganglion and dental pulp in male rats and the dental pulp of male patients. Recent studies demonstrate sex differences in pain perception and the development of chronic pain (Bowles et al., 2011; Sorge et al., 2015). Further studies are needed to investigate the possibility of sex differences in Piezo1-mediated craniofacial sensation and dental pain.

## Data Availability Statement

The original contributions presented in the study are included in the article/supplementary material, further inquiries can be directed to the corresponding author.

## Ethics Statement

The studies involving human participants were reviewed and approved by Research and Ethics Committee of the Kyungpook National University Dental Hospital. Written informed consent to participate in this study was provided by the participants' legal guardian/next of kin. The animal study was reviewed and approved by Kyungpook National University Intramural Animal Care and Use Committee.

## Author Contributions

Study design: YB. Immunohistochemistry and electron microscopy: YC, HH, and SJ. Analysis and interpretation of the data: YK, SC, and YB. Writing of the manuscript: YB. All authors have full access to all the data in this study and take responsibility for the integrity of the data and the accuracy of the data analysis. All authors contributed to the article and approved the submitted version.

## Funding

This study was supported by the National Research Foundation of Korea (NRF) grant funded by the Korea government (MSIT, NRF-2017R1A5A2015391, NRF-2021R1A2C1007061).

## Conflict of Interest

The authors declare that the research was conducted in the absence of any commercial or financial relationships that could be construed as a potential conflict of interest.

## Publisher's Note

All claims expressed in this article are solely those of the authors and do not necessarily represent those of their affiliated organizations, or those of the publisher, the editors and the reviewers. Any product that may be evaluated in this article, or claim that may be made by its manufacturer, is not guaranteed or endorsed by the publisher.
